# How funny is ChatGPT? A comparison of human- and A.I.-produced jokes

**DOI:** 10.1371/journal.pone.0305364

**Published:** 2024-07-03

**Authors:** Drew Gorenz, Norbert Schwarz

**Affiliations:** 1 Department of Psychology, University of Southern California, Los Angeles, California, United States of America; 2 Mind & Society Center, University of Southern California, Los Angeles, California, United States of America; 3 Marshall School of Business, University of Southern California, Los Angeles, California, United States of America; University of Macerata: Universita degli Studi di Macerata, ITALY

## Abstract

Can a large language model produce humor? Past research has focused on anecdotal examples of large language models succeeding or failing at producing humor. These examples, while interesting, do not examine ChatGPT’s humor production abilities in ways comparable to humans’ abilities, nor do they shed light on how funny ChatGPT is to the general public. To provide a systematic test, we asked ChatGPT 3.5 and laypeople to respond to the same humor prompts (Study 1). We also asked ChatGPT 3.5 to generate humorous satirical headlines in the style of *The Onion* and compared them to published headlines of the satirical magazine, written by professional comedy writers (Study 2). In both studies, human participants rated the funniness of the human and A.I.-produced responses without being aware of their source. ChatGPT 3.5-produced jokes were rated as equally funny or funnier than human-produced jokes regardless of the comedic task and the expertise of the human comedy writer.

## Introduction

Are large language models (LLMs) capable of humor production? Creating humor is difficult. To be perceived as funny, jokes need to be surprising [1], but also benign and not too offensive [2, 3]. Few people receive formal training in how to create humor, but many may pick it up through hearing and seeing other jokes and recognizing patterns. Presumably, an LLM could learn through a similar process by analyzing massive amounts of data.

OpenAI’s ChatGPT is one of the fastest-growing LLMs and services in terms of users [4]. It has demonstrated exceptional performance on several tasks requiring knowledge and reasoning across many fields, including education, healthcare, text generation, human-machine interaction, and scientific research [5–9]. However, one major issue limiting the usefulness of these competencies is ChatGPT’s (and other LLMs’) proclivity to hallucinate [10–12]. Hallucinating “facts” by presenting false information as accurate limits the usefulness of LLMs in many contexts, from journalism to science, but is less of a concern for many forms of comedy and entertainment (such as satirical news), which are not held to the same accuracy standards. Despite its potential to disrupt the entertainment industry and teach researchers about the processes involved in creativity, little empirical research has addressed ChatGPT’s creative writing abilities, particularly, humor production.

The available research suggests that people don’t expect algorithms to be good at generating jokes or predicting their funniness [13]. Indeed, there are many examples of ChatGPT failing to identify utterances of humor [14, 15]. LLMs are trained to recognize, translate, predict, and generate speech, not to feel emotions. This poses unique questions about what it takes to generate humor. Without the ability to feel emotions, including those associated with appreciating a good joke, could an LLM produce good, novel jokes? If it cannot feel how funny its jokes are, would its jokes be less consistently funny than people’s jokes?

Evidence for ChatGPT’s actual abilities to produce humor has been mixed [16–18] and methodologically inconclusive. Past research reported scattered examples of the model succeeding or failing at producing humor in the eyes of the respective authors without a comprehensive evaluation of the output and without comparisons to human humor production. Hence, the largely anecdotal reports, while interesting, do not shed light on how funny ChatGPT-produced humor is to the general public, nor do they analyze ChatGPT’s humor-production abilities in comparison to humans’ abilities. To fill this gap, we explore how funny ChatGPT 3.5 is compared to humans.

### Present studies

A person’s jokes can be evaluated on the merits of their quality (i.e., how good the jokes are) and quantity (i.e., how many jokes are produced) [19]. While it is clear that LLMs can produce a high quantity of jokes on command, their quality is unknown. Hence, we examine the quality of A.I.-generated jokes in comparison to humans’ jokes in the present studies. Since greater creativity is needed in the creation of novel jokes rather than the mere reproduction of jokes, we focus solely on comparing the novel humor production of LLMs and humans.

One can assess how funny a person is through self-report questionnaires, reports from others, or performance tests (i.e., fill-in-the-blank, cartoon caption, etc.). Most humor production measures are performance tests, which are designed to capture the maximal quality with which one can produce humor spontaneously, whereas self-report and other-report measures may better capture the habitual frequency or motivation of a person to produce humor [19]. The responses to performance tests can be presented to a large audience of people to evaluate the funniness of different jokes, thus reducing reliance on a few idiosyncratic opinions. In the present studies, we test ChatGPT’s humor production against human humor production across four standardized comedic tasks. In Study 1, we compare ChatGPT’s humor production abilities to laypeople’s abilities on the same tests, and in Study 2, we compare ChatGPT’s production to the production of professional satire writers.

Because comedy and satire usually address a lay audience, we measure laypeople’s opinions instead of drawing on "comedy experts.” Research in many domains of art appreciation has shown that what experts appreciate and value differs from what general audiences appreciate and value [20–22]. Whereas lay judges draw on their affective response, experts focus on structural aspects of the artwork that laypeople may not even notice [23]. Compared to non-experts, experts show attenuated positive and negative emotional reactions to positively and negatively valenced art, respectively, and tend to appreciate negative art more [24]. Hence, we consider lay ratings of the funniness of A.I. comedy more informative for the present purposes than expert ratings. Audience responses are also more relevant than expert responses from an entertainment industry perspective–A.I.-generated humor will not threaten the livelihood of comedy writers when the audience doesn’t like the jokes.

## Study 1: ChatGPT 3.5 vs. laypeople

We compared ChatGPT’s written humor production abilities to laypeople’s abilities across three diverse tasks involving different prompts. An *acronym task*, inspired by the popular party game “Quiplash” (featured in *Jackbox Games Volume 3*) [25], asked participants to think of a string of words to complete a given acronym (e.g., “C.O.W.”) in a novel and humorous way. The task requires one to think of words that are universally funny or can be combined to form a humorous phrase. The task provides character-based limitations for the words people can generate but does not impose semantic limitations on what the jokes can be about. A *fill-in-the-blank task*, inspired by the same party game, asked people to consider a question (e.g., “A lesser talked about room in the White House: ___”) and produce a funny answer to it. These prompts provide semantic-based rather than character-based limitations. For the example prompt, one must think of a potentially humorous answer that fits an audience’s associations with 1) “name for a possible room” and 2) the White House or 3) the U.S. presidency in general. Finally, a *roast joke task* (taken from Nusbaum and colleagues [26]) asked participants to imagine themselves in a scenario where they experience something negative (e.g., a friend cooks you a meal that is horrible or disgusting) and are told to be honest and humorous in describing how bad the experience was (e.g., “Wow that was so bad, _____”). Unlike the previous two tasks, this one specifically prompts participants to create an aggressive joke. This task also closely resembles aspects of humor production in everyday life in the sense that it involves reacting to everyday scenarios in a conversational style.

## Method

The study was pre-registered. Our pre-registration, S1 File, and data are available at https://osf.io/hvtgc/?view_only=6c08679d4b9a4c8892096eb76afabd3c. Analysis was conducted in R [27].

### Ethics statement

The study was approved by the University of Southern California Institutional Review Board (APP-23-04250; Date: 8/31/2023). Data were collected via an online questionnaire and participants consented by checking a box before starting the survey.

### Humor production

#### Participants

We used the services of CloudResearch.com to recruit a broad sample of Amazon Mechanical Turk (MTturk) workers located in the United States (N = 123; mean age = 39.6 years, 41% female). Compared to regular MTurk samples, CloudResearch approved participants are more likely to pass attention checks, leave the survey window less often on easily searchable questions, provide more meaningful answers, better follow instructions, and work slowly enough to be able to read all the items [28, 29]. Participants answered comedic prompts and were paid $2.58 for participating.

Because we are interested in humor production and not humor *reproduction*, we excluded participants who self-reported copying and pasting their jokes from another source (i.e., an A.I. tool, humor website, or social media) rather than producing them themselves (N = 18), as pre-registered. This resulted in a final N = 105. Participants were recruited on a single day, 10/10/2023.

#### Materials and procedure

To evaluate humor production ability, participants completed three different humor tasks in a randomized order; each task had three items. Participants were told, “We are interested in humor. We will present you with 9 prompts. Please use your own imagination to create a new, humorous answer for each.” For the *acronym task*, we asked participants to generate one new, humorous phrase for each of the following three acronyms: “S.T.D.”, “C.L.A.P.”, and “C.O.W.” For the *fill-in-the-blank task*, we asked participants to create one humorous answer for each of the three items: “A lesser talked about room in the White House: ___”, “A remarkable achievement you probably wouldn’t list on your resume: ____”, “Worst first date activity: ____.” For the *roast joke task*, we asked participants to create a humorous, conversational response to fictional scenarios that require the respondent to describe a negative experience they just had (e.g., “Imagine that one of your friends wants your opinion on how well she sings. She sings a minute or two to demonstrate her voice, and you cringe—she might be the worst singer you’ve ever heard. When she asks, “So how was it?” you decide to be honest, so you say, “To be honest, listening to that was like ____"). These items were taken from the joke stem completion task used by Nusbaum and colleagues [26] (prompts are included in S1 File).

These tasks resulted in 945 human-produced comedic responses (105 participants x 1 response per prompt x 3 prompts x 3 tasks). After completing the humor production items, participants were asked if they copied and pasted any of their joke completions from an A.I. tool (i.e., ChatGPT or other) or from a website or social media account (i.e., the Onion, Clickhole, Reddit, etc.). They were told they would not be penalized for their honesty. Lastly, we asked participants to complete basic demographic questions such as their sex, age, and political orientation. To measure political orientation, we asked participants to summarize which position best represents their viewpoint on a 9-point Likert scale from -4 (*Extremely Left-leaning*) to 0 (*Moderate*) to 4 (*Extremely Right-leaning*).

We gave ChatGPT 3.5 the same tasks and instructions as participants, but we asked it to generate twenty (rather than one) humorous answers to each prompt, resulting in 180 A.I.-produced comedic responses (20 per prompt x 3 prompts for each task x 3 tasks).

### Humor ratings

#### Participants

To compare the funniness of the human responses to A.I. responses, we recruited a separate sample of N = 200 CloudResearch approved MTurk workers located in the United States (*M*_age_ = 40.0, 41% female). Participants were paid $1.30 for participating. No participants were excluded. A sensitivity analysis indicated that our final sample size (n = 200; two-tailed, α = .05) offers .80 power to detect an effect size (*d*) as small as 0.199. Participants were recruited on a single day, 10/30/2023.

#### Design

Each participant rated the funniness of 54 responses, namely 3 human-produced and 3 ChatGPT 3.5-produced responses to each of the three items in each of the three tasks (acronym, fill-in-the-blank, and roast joke tasks described above). The to-be-evaluated responses were randomly chosen from the total set of 105 human-produced and 20 A.I.-produced responses per prompt generated during the humor production task. We did not include information about what source produced each joke, so participants could rate the humor of each joke without any preconceived bias. Data were collected via Qualtrics. Our analyses follow a 2 (Humor producer: A.I. vs. human) x 3 (Tasks: acronym, fill-in-the-blank, roast joke) within-subjects design. Participants’ funniness ratings serve as a measure of the quality of the humor produced by their peers and ChatGPT 3.5.

#### Materials and procedure

We asked participants to rate the humor of the comedic responses of a random subset (3 human- and 3 A.I.-generated) of the responses for each of the nine items. Participants made their humor ratings on a 7-point Likert scale (0 = *Not Funny at All;* 6 = *Very Funny*). Lastly, we asked participants to complete basic demographic questions such as their sex, age, and political orientation.

## Results

As pre-registered, we tested whether A.I.-generated responses were more or less funny than human-generated responses (a) by comparing participants’ mean funniness ratings for the responses and (b) by assessing how many participants rated the A.I. responses as funnier than the human responses.

**Fig 1:**
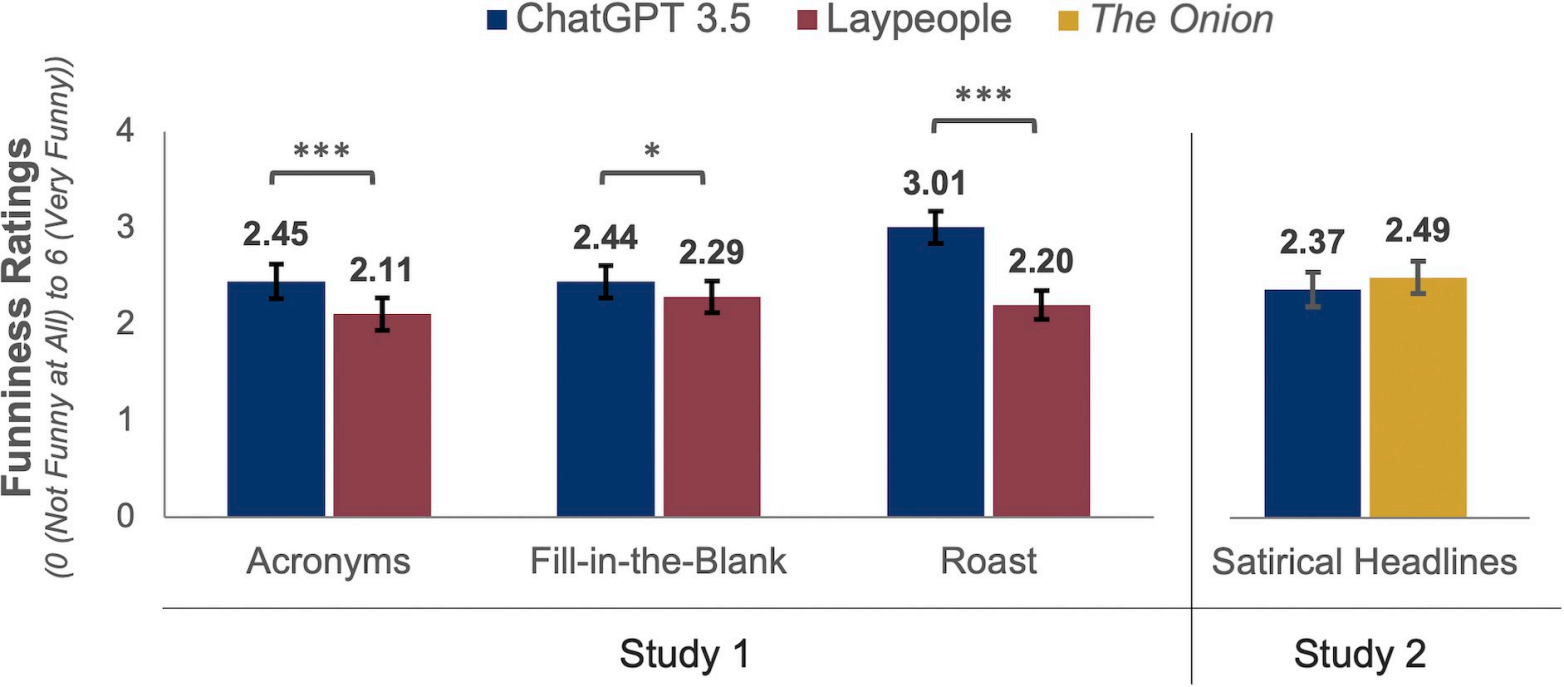
Humor appreciation ratings for ChatGPT 3.5 vs. laypeople vs. *The Onion*. Error bars represent 95% confidence intervals for mean ratings of humor. Two-tailed, paired t-test results: * *p* < .05; ** *p* < .01; *** *p* < .001.

The left-hand panel of Fig 1 shows the mean ratings by producer and type of task. Overall, participants rated the A.I.-generated responses as funnier than the human responses (*M*_*ChatGPT*_ = 2.63, 95% CI = [2.48, 2.79]; *M*_*Humans*_ = 2.20, 95% CI = [2.06, 2.34]). This held for each of the three tasks when we compared differences using paired samples t-tests (*t*_*Acronym*_(199) = 4.92, *p* < .00001; *t*_*Fill-in-the-blank*_(199) = 2.57, *p* = .0108; *t*_*Roast*_(199) = 10.78, *p* < .00001). These statistically significant differences in humor ratings across tasks are all significant with a post-hoc Bonferroni correction for the multiple analyses.

When we compare the funniness of the jokes generated by each of our human participants in the humor production task with the funniness of the jokes produced by ChatGPT 3.5, ChatGPT outperformed the majority of our human humor producers on each task. ChatGPT 3.5 performed above 73% of human producers on the acronym task, 63% of human producers on the fill-in-the-blank task, and 87% of human producers on the roast joke task.

We also explored whether the ratings showed differential agreement about the funniness of human- vs. A.I.-generated jokes. To do so, we compared the variance in ratings using the Fligner-Killeen test [30]. The variances did not significantly differ for the acronym and fill-in-the-blank tasks (*p* > .05; additional details in S1 File). However, participants showed less agreement in their ratings of the A.I.-generated than the human-generated responses to the roast joke task, resulting in a significant difference in variance (*X*^2^ (1, 200) = 4.42, *p* = .035).

In combination, these ratings indicate that our participants found ChatGPT 3.5’s responses funnier than their peers’ humor. Indeed, 69.5% of participants (95% CI = [.63, .76]) rated the A.I. responses as funnier than the human responses, which were preferred by 26.5% (95% CI = [.21, .33]); 4.0% of participants rated both as equally funny (95% CI = [.02, .08]). This majority preference for A.I.-generated responses holds for each type of task, although to different degrees (*M*_*ChatGPT*:*Acronym*_ = 58.0%, 95% CI = [.51, .65]; *M*_*ChatGPT*:*Fill-in-the-blank*_ = 50.5%, 95% CI = [.43, .58]; *M*_*ChatGPT*:*Roast*_ = 73.0%, 95% CI = [.66, .79]).

Including the demographic variables in our analyses did not change our key finding, that is, the observation that participants evaluated the A.I.-generated jokes more favorably than the human-generated jokes. We explored if demographic variables predicted any differences in participants’ humor preferences for the A.I.-generated jokes over the human-generated jokes. We regressed the total difference in humor ratings of the A.I. (vs. human)-produced jokes onto the demographic variables (i.e., sex, age, political orientation) as predictors. None of the demographic variables predicted a relative preference for A.I.-produced jokes over human-produced jokes (see table of null results in S1 File).

We also explored if demographic variables predicted any differences in participants’ humor production performance. We regressed participants’ average humor production scores onto the demographics variables (i.e., sex, age, political orientation) as predictors. Age and sex did not predict peoples’ humor production performance. People who were more right-leaning on a 9-point bipolar scale [from -4 (*Extremely Left-leaning*) to 0 (*Moderate*) to 4 (*Extremely Right-leaning*)] created jokes that were perceived as slightly less funny (*b* = -0.038, *p* = .043; additional details in S1 File).

## Discussion

Despite ChatGPT’s inability to “feel” emotions, it outperformed the average human participant across all three humor production tasks. Overall, ChatGPT 3.5 performed above 63% to 87% of human participants depending on the humor test. ChatGPT 3.5 showed particularly strong performance in the roast joke task. We found this result particularly interesting given the aggressive nature of the task. Given that ChatGPT is designed not to generate any speech that could be considered offensive or hateful, the opposite prediction could have been made.

## Study 2: ChatGPT 3.5 vs. The Onion

Given ChatGPT’s strong performance in humor production relative to laypeople, we wondered how its jokes compare to those of professional comedy writers within a commercially successful industry. For this purpose, we assessed its ability to produce satirical humor. Satire is one of the oldest types of comedy and usually involves ridiculing the vices, follies, abuses, and shortcomings of another person, group of people, or society in general. Satire can be found in many popular comedy programs from sketch comedy in *Saturday Night Live* to news satire in *The Daily Show* and *The Onion*.

We compared ChatGPT 3.5’s ability to produce humorous, satirical news headlines to that of professional comedy writers at *The Onion*. As a program with a large print following, *The Onion* boasts millions of unique monthly viewers across its website and millions of followers across social media accounts [31], making it a highly successful comedic reference for comparison. Since Study 1 suggested that demographic characteristics exert little influence on the judgments of interest in these studies, we took advantage of a convenience sample of students for Study 2.

## Method

This study was pre-registered. Our pre-registration, materials, and data can be found at https://osf.io/hvtgc/?view_only=6c08679d4b9a4c8892096eb76afabd3c. Analysis was conducted in R [27].

### Ethics statement

The study was approved by the University of Southern California Institutional Review Board (APP-23-04250; Date: 8/31/2023). Data were collected via an online questionnaire and participants consented by checking a box before starting the survey.

#### Participants

To compare the humor production of ChatGPT 3.5 to *The Onion*, we recruited N = 217 students from the University of Southern California (USC) psychology subject pool (*M*_age_ = 20.3, 62% female). No participants were excluded. A sensitivity analysis indicated that our final sample size (n = 217; two-tailed, α = .05) offers .80 power to detect an effect size (*d*) as small as 0.191. Participants were recruited from 10/20/2023 to 11/6/2023.

#### Design

We used a 2 (Source: A.I. vs. *The Onion*) within-subjects design to test humor production performance. Participants rated the funniness of 10 satirical headlines in a randomized order, 5 randomly chosen from a set of 20 headlines from *The Onion*, and 5 randomly chosen from a set of 20 headlines from ChatGPT. We did not include information about what source produced each joke, so participants could rate the humor of each joke without any preconceived bias.

#### Materials and procedure

Because ChatGPT 3.5 does not regularly receive the latest world news updates, it cannot generate satirical headlines about recent political, entertainment, or sports events. Hence, we decided to draw on *The Onion’s* ‘Local’ news section, which covers more timeless topics. This also ensures easier comparability with future replications.

We retrieved the last 50 ‘Local’ headlines published in *The Onion* before October 1, 2023 (e.g., “Man Locks Down Marriage Proposal Just As Hair Loss Becomes Noticeable” [32] and “Woman Slips Lifeguard $20 Bill To Let Her Drown” [33]) (see S1 File). We provided them to ChatGPT 3.5, asking it to produce 20 new headlines in this style. The prompt read:

“*The Onion* is a satirical news organization. They posted the below humorous, satirical news headlines under their ’Local’ news section: (… ..) Please generate 20 new humorous, satirical news article headlines similar to this style.”

The new headlines serve as the A.I.-generated stimulus set. In addition, we randomly selected 20 headlines from *The Onion’s* set of 50 published headlines as the human-generated set.

As in Study 1, we asked participants to rate the funniness of the stimuli on a 7-point scale (0 = *Not Funny at All;* 6 = *Very Funny*). Afterward, they were asked if they had previously seen any of these satirical headlines. Then, they were asked, “How much do you seek out comedy (TV, YouTube, movies, shows, standup-comedy, memes, etc.) in your life?” and “To what extent do you read or consume satirical news (e.g., the Onion, Reductress, etc.)?”; both questions were answered on a 7-point scale (0 = *Not at All;* 6 = *Very Much*). Lastly, they were asked, “Do you follow the satirical news organization, *The Onion*, on any platform?”, and if so, “Which platforms (e.g., Instagram, Facebook, etc.) do you follow *The Onion* on?” They also reported their age, gender, and political orientation. The survey was presented via Qualtrics. Written consent was obtained electronically.

The ratings for this study were collected as part of a larger survey on social media trends (all survey items are included in the open science documentation). We have no theoretical reason to assume that the other survey materials would have a differential impact on participants’ ratings of the A.I.-produced vs. human-produced satirical headlines.

## Results

As pre-registered, we tested whether the A.I.-generated satirical headlines were more or less funny than headlines generated by *The Onion’s* professional comedic writers. To do so, we (a) compared participants’ mean funniness ratings for the headlines and (b) assessed how many participants rated the A.I.-generated headlines as funnier than the professionally written headlines.

As shown in the right-hand panel of Fig 1, participants’ mean ratings did not reveal a difference in perceived funniness (*M*_*Onion*_ = 2.49, 95% CI = [2.32, 2.66] and *M*_*ChatGPT*_ = 2.37, 95% CI = [2.19, 2.55]; *t*(216) = 1.48, *p* = .14). Of the four top-rated headlines, two were generated by professional writers and two by ChatGPT, which produced the most highly rated entry (“Local Man Discovers New Emotion, Still Can’t Describe It Properly”) and the fourth most highly rated entry ("Man Achieves Personal Best in Avoiding Eye Contact With Neighbors During Awkward Elevator Ride”).

To explore whether participants agreed more when rating the funniness of *The Onion*- vs. A.I.-generated responses, we compared the variance in ratings using the Fligner-Killeen test [30]. The variances in ratings were not statistically significant (*p* > .05; additional details in S1 File).

Participants who self-reported seeking out comedy more and reading more satirical news rated the headlines as funnier (*r*_*ComedySeeking*_ = .29, 95% CI = [.16, .41]); *r*_*SatireExposure*_ = .38, 95% CI = [.26, .49]), independent of whether they were produced by A.I. or professional writers. Of the 217 participants, 22 self-reported following *The Onion* on a platform (e.g., Instagram, Facebook, website, etc.). Only 2 participants reported having seen one of the presented headlines before the study; both recognized headlines were produced by *The Onion*.

Based on their mean ratings, 48.8% (95% CI = [.42, .56]) of participants preferred *The Onion’s* headlines, whereas 36.9% preferred the A.I.-produced headlines (95% CI = [.31, .44]) and 14.3% showed no preference (95% CI = [.10, .20]). As noted, these differences were not reflected in significant mean differences in participants’ ratings.

Finally, we searched online whether any of ChatGPT 3.5’s responses were copied from somewhere else. We did not find evidence that ChatGPT merely reproduced existing headlines.

## Discussion

In sum, ChatGPT produced novel, satirical news headlines of comparable quality to *The Onion*. Participants, on average, rated the headlines as similarly funny, indicating that the average participant did not discern a difference in quality. This is particularly interesting given the high standard of comparison (i.e., professional comedy writers) in this study. If LLMs are capable of producing comparable output to professional comedians, there are large economic implications for current and aspirational comedy writers. Future research should investigate the potential of using LLMs for comedic writing across other commercially successful formats such as script writing, cartoon captioning, and meme generation once LLMs begin to incorporate image generation capabilities.

## General discussion

Humor production is difficult and highly valued. The most successful standup comedians are paid $20 million per 1-hour show taping [34]. At present, there is a free, publicly available LLM that can quickly and flexibly generate many humorous responses to many different prompts. ChatGPT 3.5, an LLM, produced jokes that were funnier than the majority of laypeople’s responses across three different tests (Study 1) and no less funny than the professional comedy writers of a major satirical news organization (Study 2).

Our studies pose several new questions. If LLMs can produce humor better than the average person, to what extent do they understand it compared to the average person? To what extent can they accurately predict how funny different jokes are for different audiences? Is the capability to feel the emotions associated with appreciating a good joke (i.e., mirth and amusement) necessary to create good jokes? Our studies suggest that the subjective experience of humor may not be required for the production of good humor–merely knowing the patterns that make up comedy may suffice. Future research may fruitfully explore this issue.

That ChatGPT can produce written humor at a quality that exceeds laypeople’s abilities and equals some professional comedy writers has important implications for comedy fans and workers in the entertainment industry. For professional comedy writers, our results suggest that LLMs can pose a serious employment threat. The implications are more positive for people who merely want to reap the benefits of elevating their everyday communications with a dose of humor. They can turn to LLMs for help. Field studies show that resumes including humorous (vs. unhumorous) self-promotion receive greater interest from professional recruiters [35]. Beginning an email with humor can result in greater trust and satisfaction in negotiations [36]. Extending these lines of investigation, future research can systematically address whether and how using appropriately tailored A.I.-generated humor can improve people’s success in personal and professional contexts. To facilitate the use of A.I.-generated humor, we add some recommendations for how to prompt LLMs for humorous ideas in our discussion of the limitations of our studies.

### Prompting A.I. to produce humor

Similar to people, LLMs give higher quality and more relevant responses when provided with clear and specific instructions. In the emerging field of prompt-engineering, researchers demonstrated that incorporating context, examples, explicit constraints, and role-playing requests (e.g., "act as my nutritionist and give me tips about a balanced Mediterranean diet") can enhance the relevance and quality of LLM responses [37, 38]. To achieve optimal output from both A.I. and people in our studies, we provided clear instructions with specific situational prompts that included semantic or character constraints. These prompts contained several high-quality joke examples for the acronym and fill-in-the-blank tasks in Study 1 and 50 joke examples from *The Onion* in addition to the data ChatGPT already had from *The Onion’s* public webpage in Study 2. We caution that LLMs may produce lower quality jokes in response to more ambiguous prompts lacking in examples, constraints, or social context. For example, Jentzsch and Kersting [17] asked ChatGPT the same prompt, “Can you tell me a joke, please?” with only subtle variations in wording (e.g., "I would love to hear a joke.") one-thousand times. As may be expected, ChatGPT returned many redundant jokes of poor quality in response to these generic prompts and Jentzsch and Kersting [17] concluded that ChatGPT “is fun, but it is not funny.” Future research can systematically explore which prompt strategies help A.I. and people produce better jokes. For example, is ChatGPT funnier when it is first asked to imagine being a famous comedian? And to what extent do prompt strategies that improve LLM comedic output also improve human comedic output? Is ChatGPT a good testing ground for learning about prompts that can improve humans’ jokes?

The content of jokes is only one aspect of comedy; another key aspect is delivery. “Text-only” jokes are often rated as less funny than jokes delivered in other formats (i.e., image and text, audiovisual) [39]. Who delivers the joke also matters. Nabi and colleagues [40] found that the same “text-only” jokes were rated as funnier when they were attributed to a famous comedian, Chris Rock, possibly because that attribution allowed readers to fill in imagined details of delivery. While ChatGPT seems to perform better than most people with the writing component of humor production, recipients’ humorous experience while reading the jokes may fall short of the experience offered by jokes that are delivered in richer formats, such as audiovisual jokes (e.g., sketch, improv, live-action, recorded videos of standup comedy) or memes (i.e., image and text), particularly when those jokes are attributed to and delivered by familiar and reputable comedy sources. At present, LLMs are severely limited in their delivery, which is why we restricted the present studies to “text-only” jokes. However, rapidly advancing A.I. technologies are bridging this gap by providing human-like text-readers that can synthesize voices, including those of professional comedians, with few data inputs [41]. Presently, someone could use an LLM to write a joke and another A.I. tool to deliver it in a familiar comedian’s voice. In the near future, these two steps may be consolidated into one, as some newer models of LLMs are developing voice-mimicking and image-generation capabilities (e.g., GPT-4o) [42–44]. These developments promise new avenues for exploring the delivery of comedic content through humans and AI.

ChatGPT has arrived at its present performance within a very short time. Updated versions of the same model have shown exponential improvements in tasks involving knowledge and analytical reasoning [45] and several competitive alternatives have already emerged with variations in their data inputs and functioning. To better understand the current trajectory of LLM humor production performance, future research could compare the performance of competing models and track whether they are getting better over time.

## Supporting information

S1 File (DOCX)
