# Supplementary material for: How funny is ChatGPT? A comparison of human- and A.I.-produced jokes
Source: PLoS One. 2024 Jul 3;19(7):e0305364. doi: 10.1371/journal.pone.0305364 (PMC11221738; doi:10.1371/journal.pone.0305364)
Supplement: S1 File — (DOCX) [file pone.0305364.s001.docx]

S1 File.

https://osf.io/hvtgc/?view_only=6c08679d4b9a4c8892096eb76afabd3c
